# Impaired activation of STAT5 upon IL-2 stimulation in Tregs and elevated sIL-2R in Sjögren’s syndrome

**DOI:** 10.1186/s13075-022-02769-y

**Published:** 2022-05-07

**Authors:** Magdalena Keindl, Richard Davies, Brith Bergum, Johan G. Brun, Daniel Hammenfors, Roland Jonsson, Valeriya Lyssenko, Silke Appel

**Affiliations:** 1 Broegelmann Research Laboratory, Department of Clinical Science, University of Bergen, 5020 Bergen, Norway; 2 Center for Diabetes Research, Department of Clinical Science, University of Bergen, Bergen, Norway; 3 NORMENT, Department of Clinical Science, University of Bergen, Bergen, Norway; 4 Flow Cytometry Core Facility, Department of Clinical Science, Faculty of Medicine, University of Bergen, Bergen, Norway; 5 Department of Rheumatology, Haukeland University Hospital, Bergen, Norway; 6 Department of Clinical Science, University of Bergen, Bergen, Norway

**Keywords:** CD25, IL2RA, Immune tolerance, Phospho-flow cytometry, Regulatory T cells

## Abstract

**Background:**

Interleukin-2 (IL-2) and the high-affinity IL-2 receptor (IL-2R) are essential for the survival of regulatory T cells (Tregs) which are the main players in immune tolerance and prevention of autoimmune diseases. Sjögren’s syndrome (SS) is a chronic autoimmune disease predominantly affecting women and is characterised by sicca symptoms including oral and ocular dryness. The aim of this study was to investigate an association between IL-2R and Treg function in patients with SS of different severity defined by the salivary flow rate.

**Methods:**

In a cross-sectional study, we determined plasma soluble IL-2R (sIL-2R) levels in women with SS (*n*=97) and healthy females (*n*=50) using ELISA. A subset of those (*n*=51) was screened for Treg function measured by the STAT5 signalling response to IL-2 using phospho-flow cytometry.

**Results:**

We found that elevated plasma levels of sIL-2R were positively associated with the severity of SS reflected by a pathologically low salivary flow. Phospho-flow analysis revealed that patients with SS have a significantly lower frequency of pSTAT5^+^ Tregs upon IL-2 stimulation compared with healthy individuals, while the frequency of Tregs and pSTAT5 in conventional T cells remained unchanged. In addition, we observed more pSTAT5^+^ Tregs at baseline in patients with SS, which is significantly associated with seropositivity and elevated sIL-2R.

**Conclusions:**

Our data indicates that Tregs have a weakened immunosuppressive function in patients with SS due to impaired IL-2/IL-2R signalling capacity. This could mediate lymphocytic infiltration into salivary glands inducing sicca symptoms. We believe that sIL-2R could act as a useful indicator for SS and disease severity.

**Supplementary Information:**

The online version contains supplementary material available at 10.1186/s13075-022-02769-y.

## Introduction

Interleukin-2 (IL-2) is a central cytokine in the maintenance of immune homeostasis due to its ability to stimulate proinflammatory effector T cells to drive immunity and its essential role in immune tolerance [1]. Regulatory T cells (Tregs) are the main players in immune tolerance and highly express the high-affinity form of the IL-2 receptor (IL-2R) consisting of three subunits, IL-2Rα (CD25), IL-2Rβ, and the common gamma chain (γc) [1]. When IL-2 binds to IL-2R a signalling cascade is induced which leads to the phosphorylation and activation of the transcription factor signal transducer and activator of transcription 5 (STAT5) [2]. Upon binding of IL-2 to IL-2R, parts of the receptor can be proteolytically cleaved, which leads to the release of IL-2Rα into the extracellular space where it can be detected as soluble IL-2R (sIL-2R) [3, 4]. Elevated plasma or serum sIL-2R has been observed in many autoimmune diseases, including type 1 diabetes (T1D) [5–7] and multiple sclerosis (MS) [8, 9]. In general, defects in IL-2 signalling and thereby Treg dysfunction is critical in the development of autoimmunity [1].

Sjögren’s syndrome (SS) is a chronic rheumatic autoimmune disorder, characterised by lymphocytic infiltrates in the salivary and lacrimal glands, leading to oral (xerostomia) and ocular dryness (keratoconjunctivitis sicca) [10]. Several studies have shown that IL-2 and IL-2R are central in the development and progression of SS. Serum and salivary sIL-2R are significantly elevated in patients with SS and are considered a disease activity index [11–17]. Furthermore, a disruption of IL-2 signalling in CD25 knock-out mice resulted in age-dependent Sjögren’s syndrome-like autoimmune lacrimal-keratoconjunctivitis [18]. In addition, peripheral blood leukocytes from patients with SS have constitutively activated STAT5 [19].

It has been suggested that it is not the number of Tregs but rather the function of Tregs that is the driving factor of autoimmune diseases [20]. Furthermore, the biological relevance of sIL-2R has not yet been unravelled; however, high sIL-2R might reflect an imbalance in Treg and effector T cell activity [7, 21, 22].

In this study, we aimed to investigate the function of Tregs from patients with SS by measuring their IL-2 signalling efficacy. Additionally, we examined whether Treg function associates with plasma levels of sIL-2R and clinical parameters.

## Methods

### Study design

In this study, we included samples from the SS biobank at the Broegelmann Research Laboratory, University of Bergen, Norway. All patients included in this investigation (*n*=97) fulfilled the 2016 American College of Rheumatology/European League Against Rheumatism classification criteria for SS [23]. We carefully selected female participants who did not suffer from any additional autoimmune disease that is associated with elevated sIL-2R. Subsequently, blood samples were collected from healthy age- and sex-matched volunteers (*n*=50) at the Broegelmann Research Laboratory, University of Bergen, Norway. The period of recruitment was from January 2017 to December 2020. The regional ethical committee approved the study (#2009/686), and all participants provided their written informed consent to participate in this study. Study participants were not involved in the design, or conduct, or reporting, or dissemination plans of our research.

We grouped patients with SS into two groups by their stimulated salivary flow. Normal/high salivary flow was categorised as >3.5 ml/5 min (range: 3.60–15.75ml/5 min) and defined as non-severe SS, whereas low/pathologic salivary flow was categorised as ≤3.5 ml/5 min (range: 0.23–3.36 ml/5 min) and defined as severe SS. In patients with SS, saliva production was stimulated by chewing on paraffin wax for 30 s. Over the course of 5 min, the saliva was subsequently collected and weighed.

### Blood sampling

The peripheral blood from patients and healthy volunteers was collected in lithium-heparin tubes (BD Diagnostics; Cat# 367526). Peripheral blood mononuclear cells (PBMCs) and plasma were isolated by density gradient centrifugation on Lymphoprep™ (STEMCELL Technologies; Cat# 07811) and cryopreserved in 50% X-vivo™ 20 (Lonza; Cat# BE04-448Q), 42.5% Profreeze™-CDM™ NAO chemically defined freeze medium (Lonza; Cat# BEBP12-769E), and 7.5% DMSO (Sigma-Aldrich; Cat# D2650) as described previously [24]. Cells were stored at −150°C and plasma was stored at −80°C.

### Enzyme-linked immunosorbent assay

We measured sIL-2R in the plasma of all selected individuals using the Human sIL-2R Instant ELISA Kit (Invitrogen; Cat# BMS212INST; assay range 0.31–20 ng/ml) according to the manufacturer’s instructions. The pre-coated plates were incubated for 3 h with the respective sample in a 1:10 dilution in sample diluent and washed 3 times. Subsequently, we added TMB substrate solution for 10 min at room temperature (RT) and stopped the reaction with the provided Stop Solution. The colour intensity was measured at 450 nm on a Emax microplate reader (Molecular Devices). All samples were measured in duplicates and plated in a randomised order. Three in-house control samples were run on each plate to investigate inter-assay variations (sample 1: values below detection rate; sample 2: CV=13.23%; sample 3: CV=25.88%). The mean value of the duplicates was used for further analysis, and the overall intra-assay variation was at CV=16.47%.

### Phospho-flow cytometry

The monoclonal antibodies and amine dyes used during the flow cytometry protocol are listed in Table S1. For the analysis of pSTAT5 in PBMCs, 51 individuals (*n*=17 per subgroup) were randomly selected from our cohort.

The selected cryopreserved PBMCs were rapidly thawed in a water bath set to 37 °C, and pre-warmed X-vivo™ 20 (Lonza; Cat# BE04-448Q) containing Pierce™ universal nuclease (25 U/ml; Thermo Scientific; Cat# 88700) was added in a dropwise fashion. The cells were washed by centrifugation at 300x*g* for 7 min at RT. The PBMCs were then resuspended in warm X-vivo™ 20 and rested in the incubator for 90 min (37 °C, 5% CO_2_). After resting, the cells were washed again by centrifugation and counted, and the cell concentration was adjusted to 5 x 10^6^ cells/ml. Subsequently, 200 μl of the cell suspension (10^6^ cells) was added per well and stimulated with 100 ng/ml human recombinant IL-2 (Immunotools; Cat# 11340023) in X-vivo™ 20 or X-vivo™ 20 only (unstimulated control) for 15 min at 37 °C. The cells were fixed with 0.5% Pierce™ PFA (Thermo Scientific; Cat# 28908) in PBS for 10 min at RT to stabilise the phosphorylation state. The cells were then washed at 350x*g* for 7 min at 4 °C. The cell pellets were then resuspended in FACS-buffer (PBS containing 0.5% BSA) and blocked for 10 min at RT with 0.5 μl FcR blocking reagent (Miltenyi Biotec, Cat# 130-059-901) per 10^6^ cells. The cells were then stained with anti-CD25 and CD127 antibodies (BD Biosciences) for 30 min at RT.

Following, the cells were washed twice with FACS-Buffer at 350x*g* for 5 min at 4 °C. The cells were resuspended in FACS-Buffer and further fixed with 1.6% PFA in PBS for 10 min at RT. The fixative was removed by centrifugation at 1000x*g* for 10 min at 4 °C and aspiration, and the cell pellets were resuspended in 10 μl PBS and chilled on ice for 10 min prior to permeabilisation. The cells were permeabilised in ice-cold 100% methanol (Sigma-Aldrich, Cat# 322415) added dropwise under a light vortex and incubated on ice for 30 min. Subsequently, the cells were washed twice with PBS at 1000x*g* for 8 min at 4 °C. Each sample was then resuspended in PBS and incubated with the respective barcode for 30 min on ice. The barcodes consisted of different combinations of Alexa Fluor 488, Pacific Blue and Pacific Orange.

Following barcoding, cells were washed twice with FACS-Buffer at 1000x*g* for 5 min at 4 °C and combined into one tube. The cells were then stained with CD3, CD4 and pSTAT5 antibodies for 30 min at RT. The samples were subsequently washed twice (1000x*g*, 5 min, 4 °C) and resuspended in FACS-buffer prior to analysis at the flow cytometer.

Samples were acquired on a LSRI Fortessa flow cytometer (BD Biosciences) with BD FACSDiva™ Software (BD Biosciences) at the Bergen Flow Cytometry Core Facility, University of Bergen, Norway. The flow cytometer was equipped with 407, 488, 561, and 635 nm lasers, and emission filters for PerCP-Cy5.5 (Long Pass (LP): 685, Band Pass (BP): 695/40), Alexa Fluor 488 (LP: 505, BP: 530/30), PE-Cy7 (LP: 750, BP: 780/60), PE (LP: -, BP: 582/15), APC (LP: -, BP: 670/14), Pacific Blue (LP: -, BP: 450/50), Pacific orange (LP: 570, BP: 585/42) and BV786 (LP: 750, BP: 780/60). The cytometer was routinely calibrated with BD cytometer setup and tracking beads (BD Biosciences, Cat# 655051). Compensation beads (Invitrogen, Cat# 01-1111-42) stained with the respective antibody were used as controls to calculate the compensation matrix. Flow cytometry data was analysed in FlowJo™ 10 (Tree Star). The representative gating strategy is shown in Fig. S1.

### Statistical analysis

Comparisons between groups, correlations and the production of associated graphs were done using R Studio (Version 1.1.456). In the phospho-flow analysis, samples were excluded from the analysis when <100 Tregs (*n*=3) were recorded and when technical errors occurred (*n*=2). Outlier values above the ordinary range were removed by *mean* ± 4 × *SD* (plasma: *n*=1; phospho-flow: *n*=0).

Mann-Whitney *U* test was used in the comparison between groups. Multiple linear regression was applied to adjust *p*-values for potential covariates. To evaluate the association between two variables we applied the Spearman correlation formula. Differences were considered statistically significant when *p*<0.05. The study was of an exploratory nature and hence no correction was made for multiple comparisons.

## Results

### Increased plasma sIL-2R levels in patients with Sjögren’s syndrome

In this study, we determined plasma sIL-2R in 97 patients with SS and 49 healthy controls. One patient sample was considered a statistical outlier and removed from further analysis. An overview of the clinical characteristics of all individuals is provided in Table 1. All study participants were female, and patients were significantly older than healthy controls (Kruskal-Wallis test: *p*=0.0036). Therefore, multiple linear regression was applied and adjusted for the age covariate in the comparison between groups.

**Table 1:** Clinical characteristics of study participants (ELISA)

	Healthy	Non-severe	Severe	*p*-value
*n*	49	42	54	-
Age (years)	50.6±8.3	54.1±17.8	58.3±15.3	0.26
Sex (% female)	100%	100%	100%	NA
Duration from onset (years)	-	8.1±10.4^a^	12.4±13.8^b^	0.22
Duration from diagnosis (years)	-	5.7±7.4^c^	8.7±7.7^d^	0.092
BMI	-	25.7±4.3^e^	25.5±5.5^f^	0.59
Stimulated salivary flow rate	-	7.0±3.3 ml/5 min	1.8±0.8 ml/5 min	< 2.2 × 10^-16^
Schirmer right (mm/5 min)	-	8.1±10.5^g^	6.3±9.0^d^	0.49
Schirmer left (mm/5 min)	-	9.4±11.3^g^	6.9±9.6^d^	0.36
Anti-SSA	-	27/73.0%^h^	35/74.5%^i^	0.88
Anti-SSB	-	8/21.6%^h^	14/29.8%^i^	0.40
Seropositive (SSA and/or SSB)	-	27/73.0%^h^	35/74.5%^i^	0.88
cDMARD	-	11/32.4%^e^	10/24.4%^j^	0.21
bDMARD (anti-TNF)	-	0^k^	0^k^	NA
Glucocorticoid	-	4/11.8%^e^	2/5.0%^l^	0.30
Cyclosporine eye drops	-	6/27.3%^m^	725.9%^n^	0.93
Oral pilocarpine	-	3/14.3%^o^	4/16%^p^	0.89
Natural tears	-	6/25%^q^	10/38.5%^r^	0.32
Angina pectoris	-	1^r^	0^l^	0.23
Autoimmune gastritis	-	0^r^	1^s^	0.43
Autoimmune thyroid disease	-	1^j^	4^t^	0.27
Proteinuria	-	0^p^	3^u^	0.16
Hematuria	-	0^p^	1^g^	0.42
Gynaecologic manifestation	-	1^p^	2^s^	0.85
Non-specific interstitial pneumonia	-	0^q^	1^h^	0.44
Fibromyalgia	-	0^q^	2^s^	0.27
Anemia	-	2^p^	3^s^	0.98
Leukopenia	-	1^p^	3^s^	0.57
Neutropenia	-	1^p^	2^s^	0.85
Thrombocytopenia	-	0^p^	1^s^	0.44
Cancer	-	1^p^	1^u^	0.78
Sclerosing cholangitis	-	0^p^	1^u^	0.44
Psoriasis	-	1^j^	1^t^	0.88
Other autoimmune diseases	-	1^j^	1^t^	0.88

Values for continuous variables are presented in mean ± SD. Mann-Whitney *U* test was used in the comparison between non-severe and severe patients

^a^n=20, ^b^n=19, ^c^n=36, ^d^n=43,^ e^n=34, ^f^n=42, ^g^n=35, ^h^n=37, ^i^n=47, ^j^n=41, ^k^n=17, ^l^n=40, ^m^n=22, ^n^n=27, ^o^n=21, ^p^n=25, ^q^n=24, ^r^n=26, ^s^n=39, ^t^n=52, ^u^n=38

**Fig. 1:**
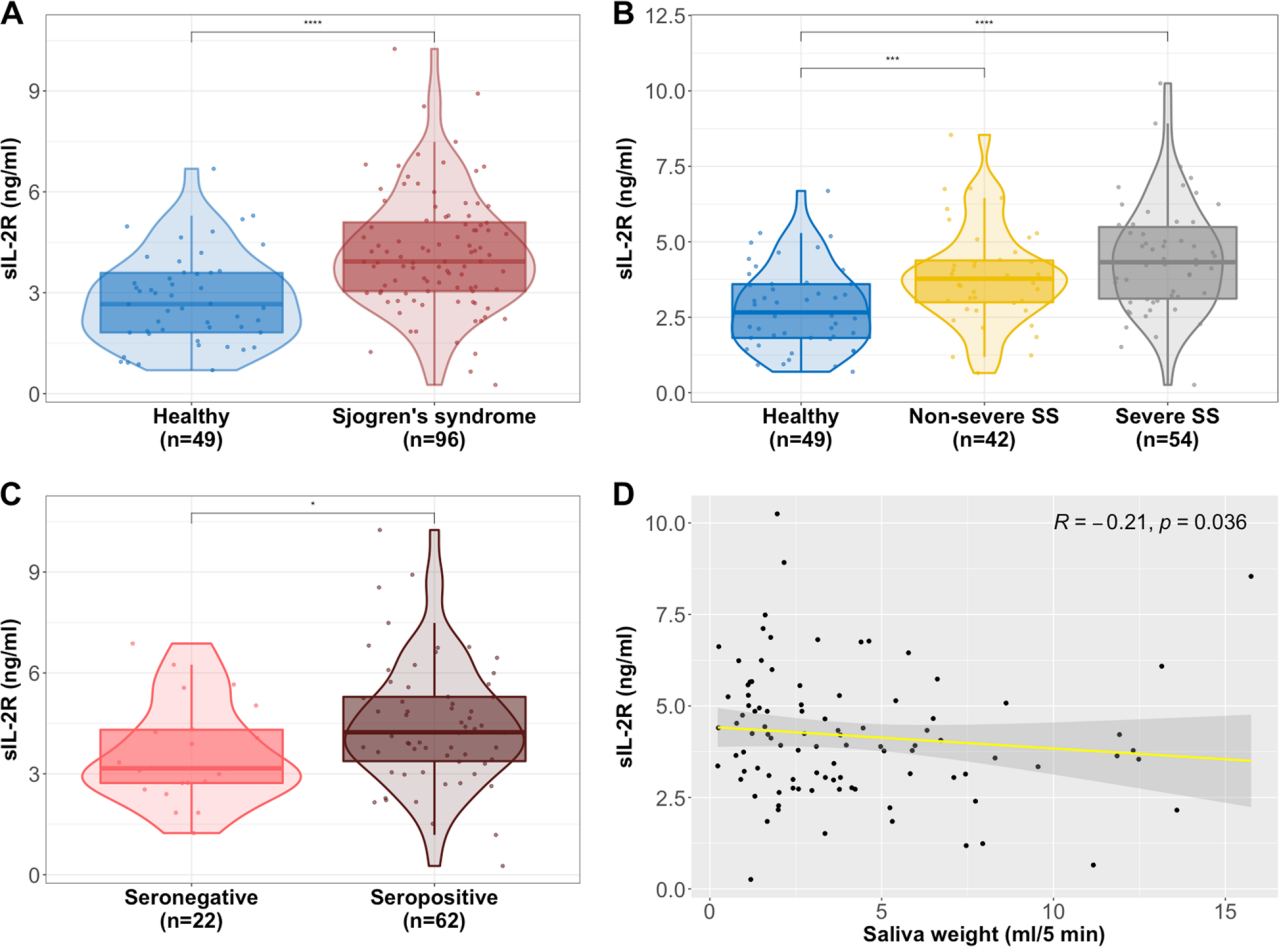
Elevated plasma sIL-2R associated with Sjögren’s syndrome. **A** Plasma sIL-2R levels were significantly increased in patients with SS compared with healthy controls. **B** Patients with low saliva production (≤3.5 ml/5 min) were considered as severe and had the highest levels of sIL-2R. Non-severe patients with SS (>3.5 ml/5 min) had significantly higher sIL-2R compared with healthy controls. **C** High plasma sIL-2R was associated with seropositivity. **D** The amount of saliva produced per min correlated negatively with plasma sIL-2R. (Mann-Whitney *U* test was used in the comparison between the different groups. To evaluate the association between two variables, we applied the Spearman correlation formula. *p≤0.05, **p≤0.01, ***p≤0.001 and ***p≤0.0001)

We observed a significant increase of sIL-2R (*p*=1.70 × 10^-6^, *p*_*adj*_=1.31 × 10^-5^) in patients with SS (total patient group) as compared to healthy individuals (Fig. 1A). This increase was gradual in relation to disease severity measured by the production of the saliva with a cut-off at 3.5 ml/5 min (healthy vs. non-severe: *p*=9.81 × 10^-4^, *p*_*adj*_=0.0011; healthy vs. severe: *p*=1.41 × 10^-6^, *p*_*adj*_=2.66 × 10^-5^; non-severe vs. severe: *p*=0.089, *p*_*adj*_=0.130) (Fig. 1B). Furthermore, higher sIL-2R was associated with seropositivity for anti-SSA and/or anti-SSB (*p*=0.045, *p*_*adj*_=0.033) (Fig. 1C). This association was strongest in non-severe SS with a normal/high salivary flow (non-severe: *p*=0.030, *p*_*adj*_=0.021; Severe: *p*=0.57, *p*_*adj*_=0.38) (Fig. S2A). In patients with SS, the level of sIL-2R correlated negatively with the amount of the saliva produced (*R*= −0.21, *p*=0.036) (Fig. 1D). The distribution of saliva weight produced by patients with SS is displayed in Fig. S2B.

### Phosphorylation of STAT5 in Sjögren’s syndrome

**Fig. 2:**
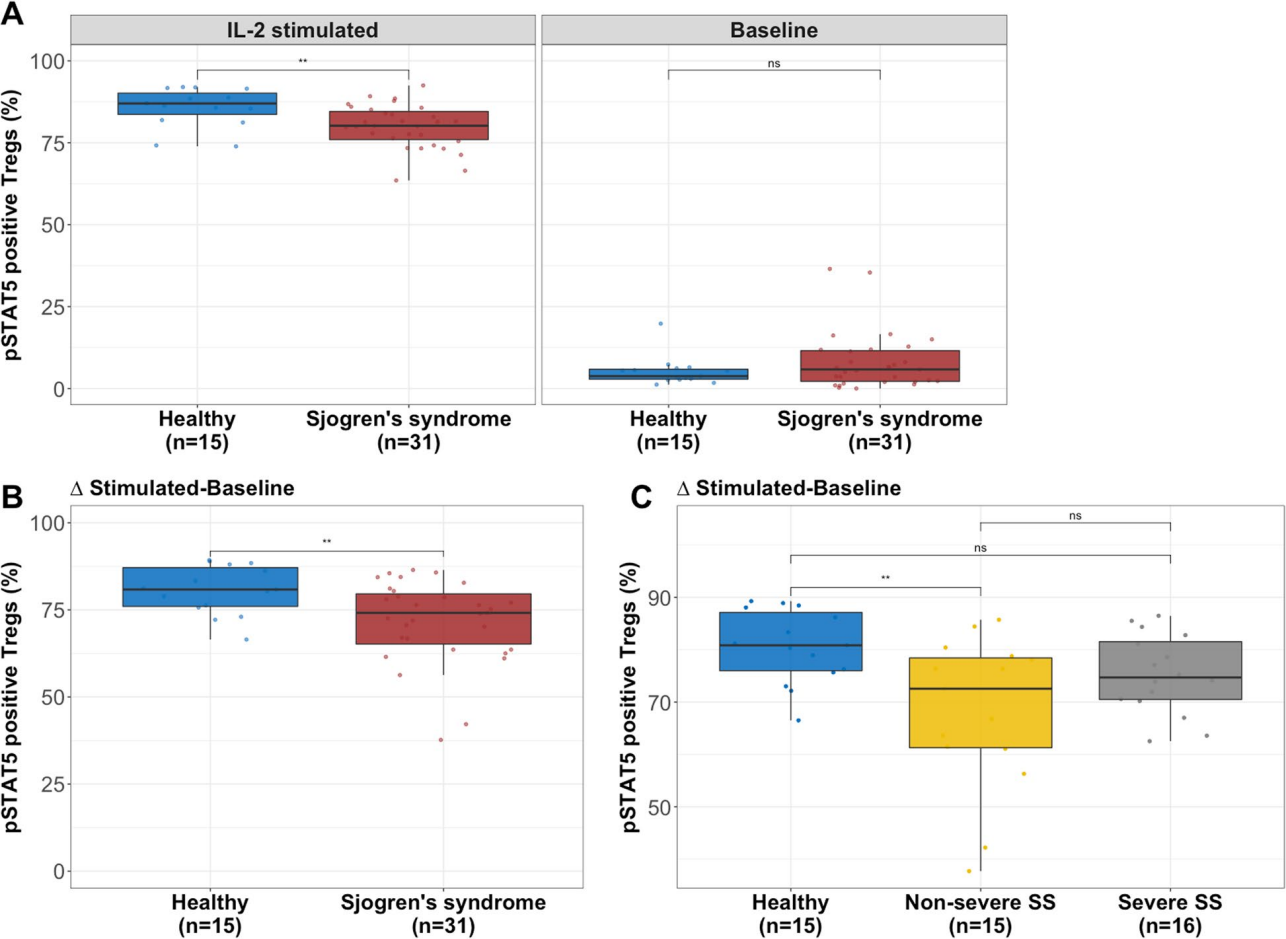
Reduced pSTAT5 upon IL-2 stimulation associated with Sjögren’s syndrome. **A** Patients with SS had significantly lower frequency of pSTAT5^+^ Tregs upon IL-2 stimulation (left panel) and higher pSTAT5^+^ Tregs at baseline (right panel). **B** The difference in pSTAT5^+^ Tregs between baseline and IL-2 stimulation was significantly lower in patients with SS. **C** The delta in pSTAT5+ Tregs was lowest in patients with non-severe SS. (Mann-Whitney *U* test was used in the comparison between the different groups. *p≤0.05, **p≤0.01, ***p≤0.001 and ***p≤0.0001)

Phosphorylation of STAT5 in Tregs from healthy controls and patients with SS was investigated by phospho-flow cytometry. For this analysis, we randomly selected 51 individuals representative of each subgroup in regard to sIL-2R (Fig. S3A). Clinical characteristics for this cohort subset are provided in Table 2. We identified age, day of analysis and seeded cell concentration as significant covariates, hence we used multiple linear regression to adjust *p*-values for those in the comparison between groups. Because of the bimodal STAT5 phosphorylation response to IL-2 in Tregs (Fig. 2E), we reported response to IL-2 as the frequency of pSTAT5^+^ Tregs rather than geometric median fluorescence intensity [25].

**Table 2:** Clinical characteristics of study participants (pSTAT5)

	Healthy	Non-severe	Severe	*p*-value
*n*	15	15	16	-
Age (years)	50.3±9.0	59.7±17.2	61.9±17.0	0.66
Sex (% female)	100%	100%	100%	NA
Duration from onset (years)	-	5.7±10.2^a^	14.8±10.9^a^	0.064
Duration from diagnosis (years)	-	5.7±7.0	9.8±7.2^b^	0.079
BMI	-	25.6±4.6^b^	25.3±6.2^b^	0.88
Stimulated salivary flow rate	-	7.2±3.1 ml/5 min	1.5±0.9 ml/5 min	6.66 × 10^-9^
Schirmer right (mm/5 min)	-	8.0±9.3	5.5±5.9^b^	0.69
Schirmer left (mm/5 min)	-	7.9±7.4	6.5±7.0^b^	0.61
Anti-SSA	-	11/73.3%	13/86.7%^c^	0.39
Anti-SSB	-	2/13.3%	5/33.3%^c^	0.21
Seropositive (SSA and/or SSB)	-	11/73.3%	13/86.7%^c^	0.39
cMARD	-	5/35.7%^d^	4/33.3%^e^	0.93
bMARD (anti-TNF)	-	0/0.0%^f^	0/0.0%^a^	NA
Glucocorticoid	-	3/21.4%^d^	1/9.1%^g^	0.44
Cyclosporine eye drops	-	3/37.5%^h^	3/33.3%^f^	0.91
Oral pilocarpine	-	1/14.3%^i^	1/12.5%^h^	1
Natural tears	-	4/40.0%^j^	2/28.6%^i^	0.68
Angina pectoris	-	1^b^	0^e^	0.38
Autoimmune thyroid disease	-	0	2	0.18
Gynaecologic manifestation	-	1^b^	0^e^	0.38
Anemia	-	2^b^	0^e^	0.18
Leukopenia	-	1^b^	1^e^	1
Neutropenia	-	1^b^	0^e^	0.38
Cancer	-	1^b^	1^e^	1

Values for continuous variables are presented in mean ± SD. Mann-Whitney U test was used in the comparison between non-severe and severe patients

^a^n=6, ^b^n=13, ^c^n=15, ^d^n=14, ^e^n=12, ^f^n=9, ^g^n=11, ^h^n=8, ^i^n=7, ^j^n=10

**Fig. 3:**
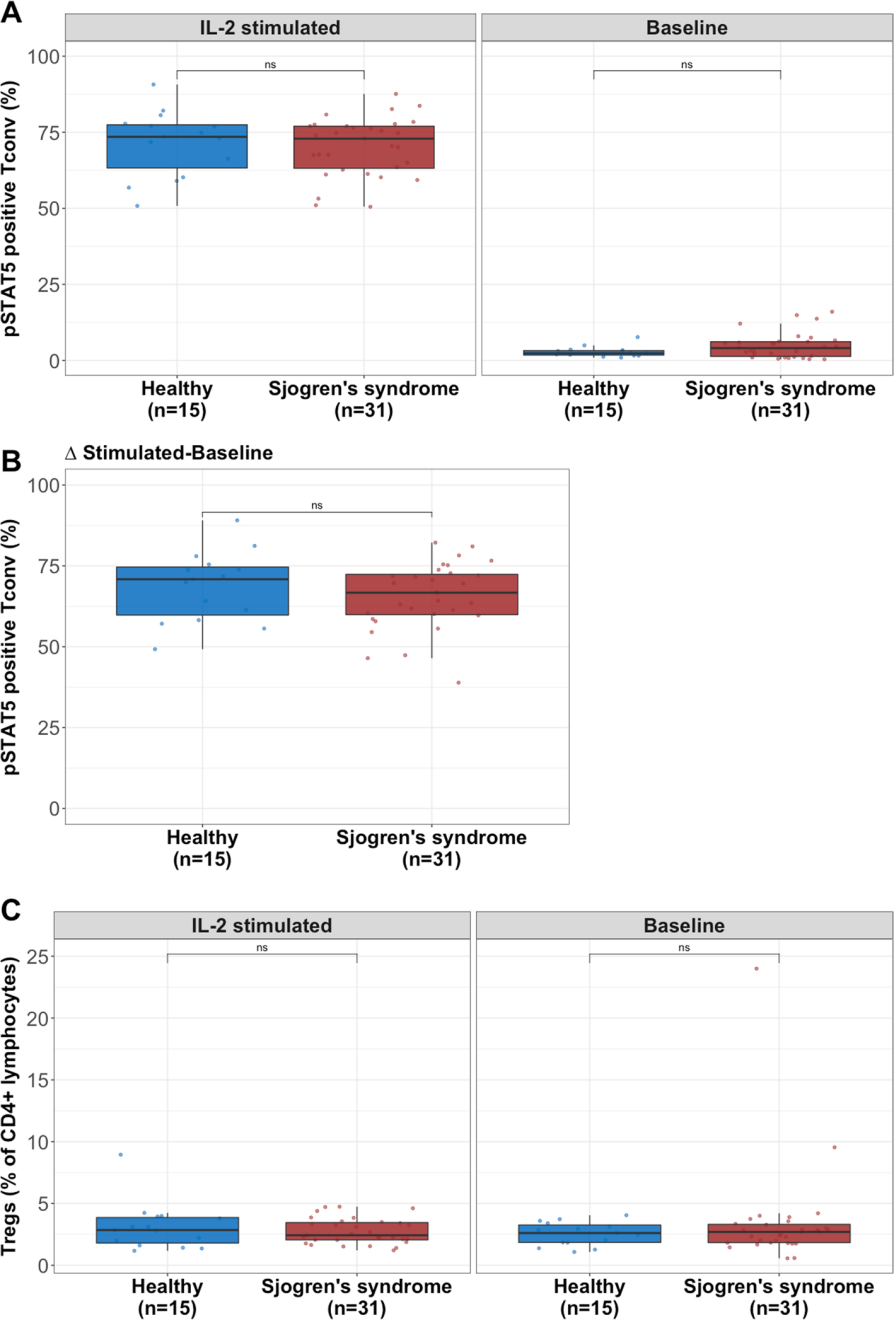
Similar frequency of pSTAT5^+^ Tconv and Tregs between patients and controls. **A** Patients with SS had similar frequency of pSTAT5^+^ Tconv upon IL-2 stimulation (left panel) and at baseline (right panel) as compared to healthy controls. **B** No difference in pSTAT5^+^ Tconv between patients and controls was observed when analysing the difference between IL-2 stimulated and baseline. **C** There was no difference in Treg frequency between healthy individuals and patients with SS. (Mann-Whitney *U* test was used in the comparison between the different groups. *p≤0.05, **p≤0.01, ***p≤0.001 and ***p≤0.0001)

**Fig. 4:**
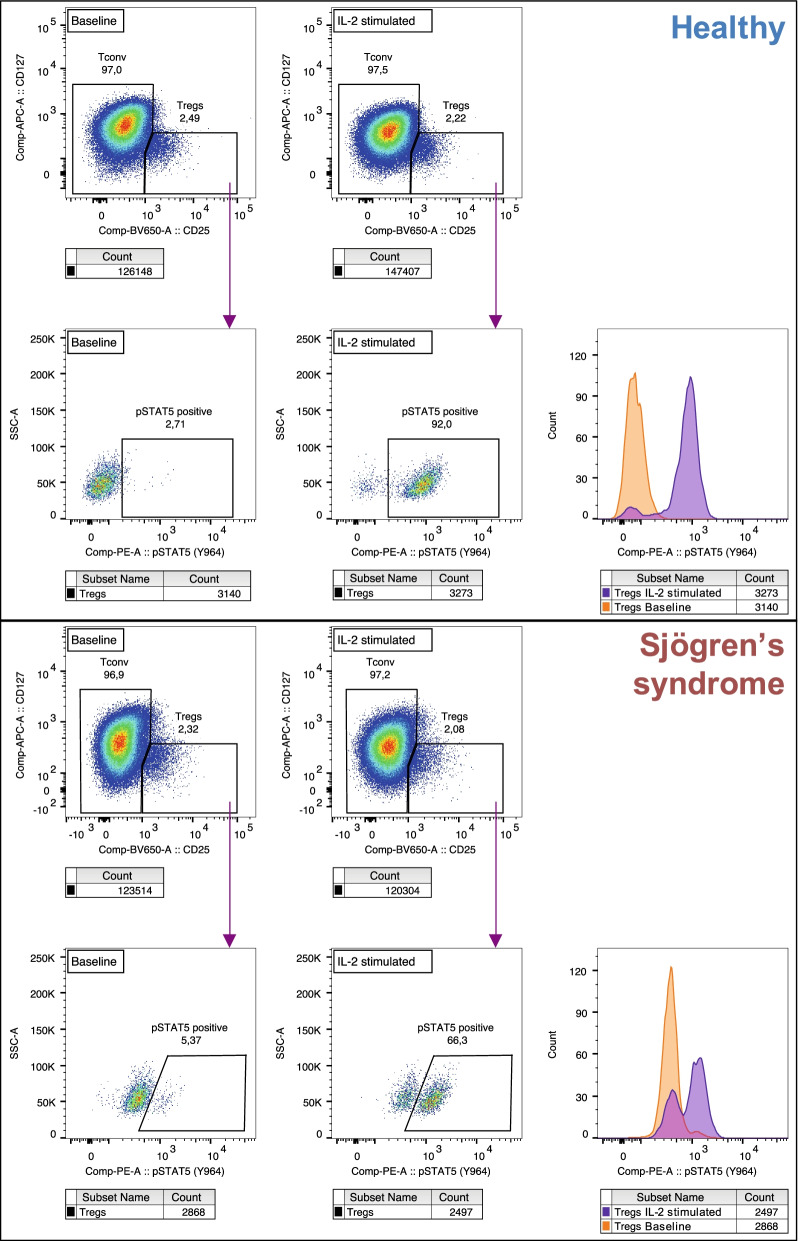
The phospho-flow cytometry results of two individual representatives of the respective subgroup are displayed. The histograms clearly display the bimodal STAT5 phosphorylation response to IL-2 in Tregs. Tregs from patients with SS achieved a lower STAT5 phosphorylation upon IL-2 stimulation compared with healthy controls, while Treg frequency remained stable

We identified a significant decrease in the frequency of pSTAT5^+^ Tregs (*p*=0.0044, *p*_*adj*_=0.041) upon IL-2 stimulation in patients with SS compared with healthy controls (Fig. 2A, left panel). Furthermore, we observed that patients with SS had a slightly higher frequency of pSTAT5^+^ Tregs (*p*=0.46, *p*_*adj*_=0.16) at baseline (without stimulation) (Fig. 2A, right panel). To correct for these differences at basal levels, we subtracted baseline from IL-2 stimulated pSTAT5^+^ Tregs for each individual. We found that healthy controls achieved a significantly higher frequency of pSTAT5^+^ Tregs (*p*=0.0093, *p*_*adj*_=0.038) compared with patients with SS (Fig. 2B). In conventional T cells (Tconv), the frequency of pSTAT5^+^ cells was similar between patients and healthy controls (IL-2 stimulated: *p*=0.7785, *p*_*adj*_=0.0411; Baseline: *p*=0.2413, *p*_*adj*_=0.160; Stimulated-baseline: *p*=0.7548, *p*_*adj*_=0.855) (Fig. 3A, B) indicating a Treg-specific effect. Interestingly, the observed decrease of STAT5 activation in Tregs in SS was most profound in non-severe SS with a normal/high salivary flow (healthy vs. non-severe: *p*=0.0086, *p*_*adj*_=0.030; healthy vs. severe: *p*=0.054, *p*_*adj*_=0.16; non-severe vs. severe: *p*=0.22, *p*_*adj*_=0.32) (Fig. 2C). In general, the frequency of Tregs was not different between healthy individuals and SS (IL-2 stimulated: *p*=0.98, *p*_*adj*_=0.47; Baseline: *p*=0.93, *p*_*adj*_=0.40) (Fig. 3C). The phospho-flow cytometry data from two individual representatives of the respective subgroup (healthy and SS) is displayed in Fig. 4.

Furthermore, we observed that a higher frequency of pSTAT5^+^ Tregs at baseline was significantly associated with seropositivity for anti-SSA and/or anti-SSB (*p*=0.0051, *p*_*adj*_=0.35) (Fig. S4A, right panel). There was no difference in the frequency of pSTAT5^+^ Tregs between seropositive and seronegative patients in the IL-2 stimulated cells (*p*=0.53, *p*_*adj*_=0.065) (Fig. S4A, left panel). When analysing the difference between IL-2 stimulated and baseline, we found that seropositive patients achieved a slightly lower frequency of pSTAT5^+^ Tregs compared with seronegative patients (*p*=0.058, *p*_*adj*_=0.061) (Fig. S4B).

Interestingly, the frequency of pSTAT5^+^ Tregs at baseline positively correlated with plasma levels of sIL-2R (*p*=0.0043, *R*=0.41) (Fig. S5A, right panel). No correlation was observed in the IL-2 stimulated cells (*p*=0.70, *R*=-0.058) (Fig. S5A, left panel). Furthermore, the delta frequency of pSTAT5^+^ Tregs (stimulated-baseline) did not significantly correlate with plasma sIL-2R (*p*=0.073, *R*=-0.27) (Fig. S5B). However, a trend could be observed where patients with the highest plasma levels of sIL-2R had the lowest delta frequency of pSTAT5+ Tregs or, in other words, the weakest IL-2/IL-2R signalling capacity in Tregs.

## Discussion

In the present study, we revealed that peripheral Tregs from patients with SS have an impaired activation of STAT5 upon IL-2 stimulation compared with healthy individuals. Further, significantly, a higher baseline frequency of pSTAT5^+^ Tregs was associated with SS and higher plasma sIL-2R. In agreement with published studies, we confirmed that plasma sIL-2R levels are significantly increased in patients with SS compared with healthy individuals. Finally, we found that patients with a more severe phenotype reflected by a pathologically low salivary flow had the highest levels of sIL-2R and that plasma sIL-2R correlated negatively with the salivary flow.

To this day, the biological relevance of sIL-2R remains unknown. Elevated levels have been suggested to reflect an imbalance in Treg and effector T cell activity [7, 21, 22]. IL-2 is an essential cytokine for both cell types, and depending on the levels of IL-2 in their microenvironment, it can favour either the one or the other. Effector T cells mostly express the intermediate affinity IL-2R which requires higher levels of IL-2 in order for the cells to respond and become activated. Tregs on the other hand constitutively express the high-affinity IL-2R giving them a benefit when lower levels of IL-2 are available and allowing them to outcompete proinflammatory effector T cells [26]. Interestingly, sIL-2R has the capacity to bind IL-2 with low affinity [27], which can have opposing effects depending on the affected cell type [26]. By binding IL-2 in the extracellular space, sIL-2R might act as a decoy-receptor reducing the bioavailability of IL-2 to favour tolerance over immunity. Increased shedding of sIL-2R by Tregs may thereby be a compensation mechanism for Tregs with an impaired function. Alternatively, sIL-2R bound to IL-2 can also benefit immunity by *in trans* presenting IL-2 to effector T cells expressing the intermediate-affinity IL-2R allowing them to boost IL-2 signalling [26]. Overall, higher sIL-2R in patients with an autoimmune disease could reflect an impaired tolerance or an increased inflammation, which can in turn be induced by an impaired tolerance.

To investigate this further, we stimulated peripheral immune cells from healthy individuals and patients with SS with IL-2 and measured phosphorylation of STAT5 downstream of the IL-2/IL-2R signalling cascade. We found that SS was associated with less pSTAT5^+^ Tregs upon IL-2 stimulation compared to healthy controls indicating a reduced IL-2 signalling efficacy. Indeed, IL-2-induced STAT5 activation has been shown to be crucial for the immunosuppressive activity of Tregs [28]. The phosphorylation of STAT5 in Tconv was not impaired in patients with SS indicating that our results are Treg-specific. Furthermore, we found no difference in the general frequency of Tregs between patients with SS and healthy controls. Taken together, this is another confirmation for the theory that it is not the number of Tregs but the function of Tregs which is central in the development and progression of autoimmune diseases [20].

It is still debatable whether elevated sIL-2R in autoimmune diseases reflects an impaired Treg function or an increased effector T cell activity. Even though we found that patients with SS had significantly higher plasma sIL-2R and a reduced frequency of pSTAT5^+^ Tregs upon IL-2 stimulation, the two variables did not significantly correlate. Further studies are necessary to elucidate the meaning of elevated sIL-2R in autoimmune diseases and unravel whether these sIL-2R molecules are cleaved from Tregs or activated effector T cells.

In our phospho-flow experiments, we found that patients with SS had a higher frequency of pSTAT5^+^ Tregs at baseline. This is in accordance with another study by Pertovaara et al. who previously reported constitutively activated STAT5 in different immune cells from patients with SS [19]. Similar results have been published on systemic lupus erythematosus (SLE) [29], lymphoid leukaemia [30] and chronic myeloid leukaemia [31]. Furthermore, constitutively activated STAT5 in SLE has been associated with lupus activity index [29]. Interestingly, a relationship between elevated sIL-2R and disease activity has also been described in multiple autoimmune diseases including SS [11, 14, 15], T1D [7], MS [8], rheumatoid arthritis [32], scleroderma [33], inflammatory myopathies [34] and various cancers [32]. In this study, we detected the highest levels of sIL-2R in seropositive patients and in patients with a more severe phenotype reflected by a pathologically low salivary flow. Additionally, we found that plasma sIL-2R positively correlated with the baseline frequency of pSTAT5^+^ Tregs. Collectively, these data indicate that constitutively phosphorylated STAT5 in SS might also be associated with disease activity and severity. Importantly, a large registry-based study from Sweden reported an increased risk of developing type 2 diabetes in a range of autoimmune diseases including SS [35]. This association was particularly strong in patients with the early-onset manifestation of diabetes before 50 years of age, which indicates a strong link between sIL-2R and potentially impaired Treg function aggravating the progression to diabetes.

Despite discovering that patients with SS have an impaired Treg function, it is difficult to determine at what step of the IL-2/IL-2R signalling cascade the signalling got interrupted. Moreover, different genetic predispositions and proteins could disturb the signalling in the individual patients. Phosphatase tyrosine-protein phosphatase non-receptor type 2 (PTPN2) is a ubiquitously expressed negative regulator of the IL-2/IL-2R signalling cascade and has been investigated in different autoimmune diseases [2, 21]. Genetic polymorphisms in the *PTPN2* gene region have been linked with different autoimmune diseases such as T1D [36–38], rheumatoid arthritis [39], celiac disease [40] and Crohn’s disease [39]. Furthermore, we recently reported that SNPs in *PTPN2* are significantly associate with plasma levels of sIL-2R in T1D [7]. In addition, certain *PTPN2* SNPs correlate with impaired IL-2 signalling in CD4^+^ T cells measured by decreased pSTAT5 [41]. Potentially, PTPN2 may be involved in the observed impaired Treg function in SS as well.

A limitation of this study is the rather small sample size, which makes it difficult to draw reliable conclusions particularly when subdividing patients into even smaller groups by various clinical features. A tremendous gender bias is observed in SS with the female to male ratio being 9:1 [42]. In our study, we only included female individuals as our SS biobank did not have a sufficient sample number of male patients available. Furthermore, working with a highly sensitive analysis such as phospho-flow cytometry, small laboratory and assay variations can have drastic outcomes. To limit these variations to a minimum, we randomised the samples between analysis days and used biological barcoding to reduce staining variability, increase uniformity and shorten the total acquisition time at the flow cytometer.

Genotyping patients with SS to identify potential associations between genetic variants and sIL-2R and Treg function is of high interest. Furthermore, highlighting IL-2/IL-2R signalling as a central immune pathway in SS could lead to the identification of novel treatment targets. Low-dose IL-2 treatment has shown promising effects by expanding and activating Tregs in a number of clinical trials for several autoimmune diseases most prominently type 1 diabetes [43–45]. Potentially, low-dose IL-2 administration may be a beneficial treatment for people with SS and other rheumatic diseases. Additionally, it is of great interest to investigate an association of sIL-2R and Treg function in other autoimmune diseases to identify whether a central mechanism is at play.

## Conclusions

In summary, a reduced immunosuppressive function of Tregs due to the impaired IL-2/IL-2R signalling observed in SS could induce a more aggressive lymphocytic infiltration into salivary glands. This in turn leads to oral dryness which is associated with plasma sIL-2R. To conclude, sIL-2R could potentially act as a useful biomarker for SS and disease severity and thereby assist in an earlier diagnosis and treatment.

## Supplementary Information

**Additional file 1: Table S1.** Fluorescently labelled antibodies and amine dyes used for phospho-flow cytometry. **Figure S1.** Representative gating strategy used in the analysis of phosphorylation of STAT5 in PBMCs. Lymphocytes were gated based on their forward scatter area (FSC-A) and side scatter area (SSC-A) properties, followed by singlet gating based on SSC-A and side scatter height (SSC-H). Subsequently, CD3+ CD4+ T cells were gated. The different samples were then identified through the intensities of their Alexa Fluor 488, Pacific Blue and Pacific Orange staining (barcoding). One representative sample is shown to display further gating steps. Tregs and Tconv were identified based on their surface antigen presentation (Tregs: CD25+ CD127- , Tconv: CD25- ). Finally, cells with phosphorylated STAT5 at position Y964 were gated in Tregs and Tconv. **Figure S2.** (A) In the non-severe Sjögren’s syndrome (SS) group seropositive patients had significantly higher sIL-2R compared with seronegative patients. (B) A histogram of the saliva weight (ml/5 min) produced by patients with Sjögren’s syndrome after paraffin stimulation. The vertical dashed line shows the cut-off at 3.5 ml/5 min which we used to split between patients with severe and non-severe phenotype. (Mann-Whitney U test was used in the comparison between the different groups. *p≤0.05, **p≤0.01, ***p≤0.001, and ***p≤ 0.0001.). **Figure S3.** The selected 51 individuals for the phospho-flow cytometry analysis are representative of the whole cohort and display significantly higher sIL-2R in patients with Sjögren’s syndrome (SS) (A), particularly those with low saliva production (B). (Mann-Whitney U test was used in the comparison between the different groups. *p ≤0.05, **p≤0.01, ***p≤0.001, and ***p≤0.0001). **Figure S4.** Percentage of pSTAT5+ Tregs and their association with serology. (A) Seropositive patients with Sjögren’s syndrome had a significantly higher frequency of pSTAT5+ Tregs at baseline compared with seronegative patients (right panel), whereas no difference was observed in IL-2 stimulated Tregs (left panel). (B) The difference in pSTAT5+ Tregs between baseline and IL-2 stimulation was slightly lower in seropositive patients with Sjögren’s syndrome. (Mann-Whitney U test was used in the comparison between the different groups. *p≤ 0.05, **p≤0.01, ***p≤0.001, and ***p≤0.0001.). **Figure S5.** Frequency of pSTAT5+ Tregs and their association with plasma sIL-2R. (A) Higher plasma sIL-2R levels significantly associated with higher frequency of pSTAT5+ Tregs at baseline (right panel), whereas no correlation was observed in IL-2 stimulated Tregs (left panel). (B) The difference in pSTAT5+ Tregs between baseline and IL-2 stimulation did not correlate with sIL-2R (To evaluate the association between two variables we applied the Pearson correlation formula.).

## Data Availability

The datasets used and/or analysed during the current study are available from the corresponding author on reasonable request.

## Abbreviations

CD: Cluster of differentiation
IL-2: Interleukin-2
IL-2R: Interleukin-2 receptor
MS: Multiple sclerosis
PBMC: Peripheral blood mononuclear cells
RT: Room temperature
sIL-2R: Soluble interleukin-2 receptor
SS: Sjögren’s syndrome
STAT: Signal transducer and activator of transcription
T1D: Type 1 diabetes
Tconv: Conventional T cells
Tregs: Regulatory T cells
