# Supplementary material for: Impaired activation of STAT5 upon IL-2 stimulation in Tregs and elevated sIL-2R in Sjögren’s syndrome
Source: Arthritis Res Ther. 2022 May 7;24:101. doi: 10.1186/s13075-022-02769-y (PMC9077945; doi:10.1186/s13075-022-02769-y)
Supplement: Supplementary file 1 — Additional file 1: Table S1. Fluorescently labelled antibodies and amine dyes used for phospho-flow cytometry. Figure S1. Representative gating strategy used in the analysis of phosphorylation of STAT5 in PBMCs. Lymphocytes were gated based on their forward scatter area (FSC-A) and side scatter area (SSC-A) properties, followed by singlet gating based on SSC-A and side scatter height (SSC-H). Subsequently, CD3+ CD4+ T cells were gated. The different samples were then identified through the intensities of their Alexa Fluor 488, Pacific Blue and Pacific Orange staining (barcoding). One representative sample is shown to display further gating steps. Tregs and Tconv were identified based on their surface antigen presentation (Tregs: CD25+ CD127- , Tconv: CD25- ). Finally, cells with phosphorylated STAT5 at position Y964 were gated in Tregs and Tconv. Figure S2. (A) In the non-severe Sjögren’s syndrome (SS) group seropositive patients had significantly higher sIL-2R compared with seronegative patients. (B) A histogram of the saliva weight (ml/5 min) produced by patients with Sjögren’s syndrome after paraffin stimulation. The vertical dashed line shows the cut-off at 3.5 ml/5 min which we used to split between patients with severe and non-severe phenotype. (Mann-Whitney U test was used in the comparison between the different groups. *p≤0.05, **p≤0.01, ***p≤0.001, and ***p≤ 0.0001.). Figure S3. The selected 51 individuals for the phospho-flow cytometry analysis are representative of the whole cohort and display significantly higher sIL-2R in patients with Sjögren’s syndrome (SS) (A), particularly those with low saliva production (B). (Mann-Whitney U test was used in the comparison between the different groups. *p ≤0.05, **p≤0.01, ***p≤0.001, and ***p≤0.0001). Figure S4. Percentage of pSTAT5+ Tregs and their association with serology. (A) Seropositive patients with Sjögren’s syndrome had a significantly higher frequency of pSTAT5+ Tregs at baseline compared with seronegativ [file 13075_2022_2769_MOESM1_ESM.pdf]

Supplemental Material

Table S1 Fluorescently labelled antibodies and amine dyes used for phospho-flow cytometry.

Marker/Dye	Conjugate	Clone	Dilution	Producer
CD3	Brilliant Violet 786™	SK7	1:300	BD Biosciences, 563800
CD4	APC-Cy™7	RPA-T4	1:160	BioLegend, 300518
CD25 (IL-2RA)	Brilliant Violet 650™	BC96	1:80	BioLegend, 302634
CD127 (IL-7RA)	Alexa Fluor® 647	A019D5	1:160	BioLegend, 351318
STAT5 (pY694)	PE	Clone 47	1:40	BD Biosciences, 562077
Alexa Fluor® 488	-	-	0, 200, 800, 3200 ng/ml	Invitrogen, A200000
Pacific Blue™	-	-	0, 680, 2500 ng/ml	Invitrogen, P10163
Pacific Orange™	-	-	0, 3200 ng/ml	Invitrogen, P30253

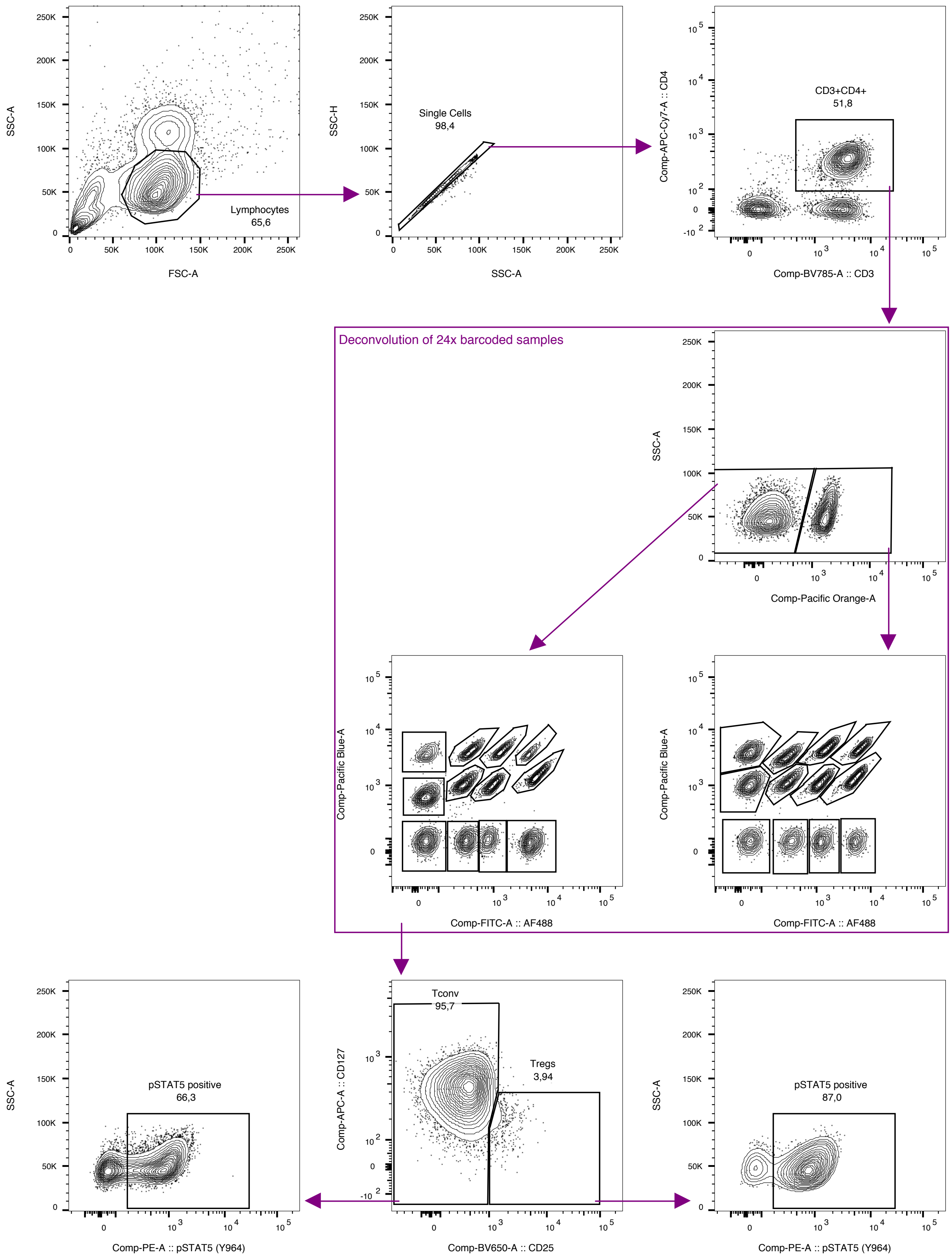

Figure S1 Representative gating strategy used in the analysis of phosphorylation of STAT5 in PBMCs. Lymphocytes were gated based on their forward scatter area (FSC-A) and side scatter area (SSC-A) properties, followed by singlet gating based on SSC-A and side scatter height (SSC-H). Subsequently, $CD3^+CD4^+$ T cells were gated. The different samples were then identified through the intensities of their Alexa Fluor 488, Pacific Blue and Pacific Orange staining (barcoding). One representative sample is shown to display further gating steps. Tregs and Tconv were identified based on their surface antigen presentation (Tregs: $CD25^+CD127^-$, Tconv: $CD25^-$). Finally, cells with phosphorylated STAT5 at position Y964 were gated in Tregs and Tconv.

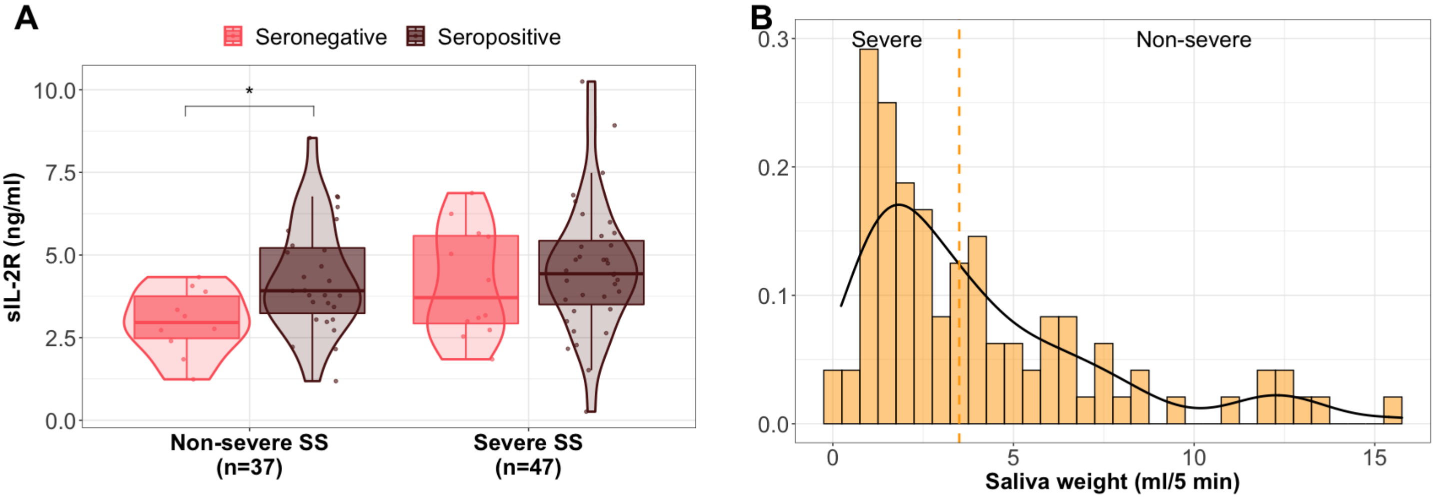

Figure S2 (A) In the non-severe Sjögren's syndrome (SS) group seropositive patients had significantly higher sIL-2R compared with seronegative patients. **(B)** A histogram of the saliva weight (ml/5 min) produced by patients with Sjögren's syndrome after paraffin stimulation. The vertical dashed line shows the cut-off at 3.5 ml/5 min which we used to split between patients with severe and non-severe phenotype. (Mann-Whitney U test was used in the comparison between the different groups. $*p\leq0.05$, $**p\leq0.01$, $***p\leq0.001$, and $***p\leq 0.0001$.).

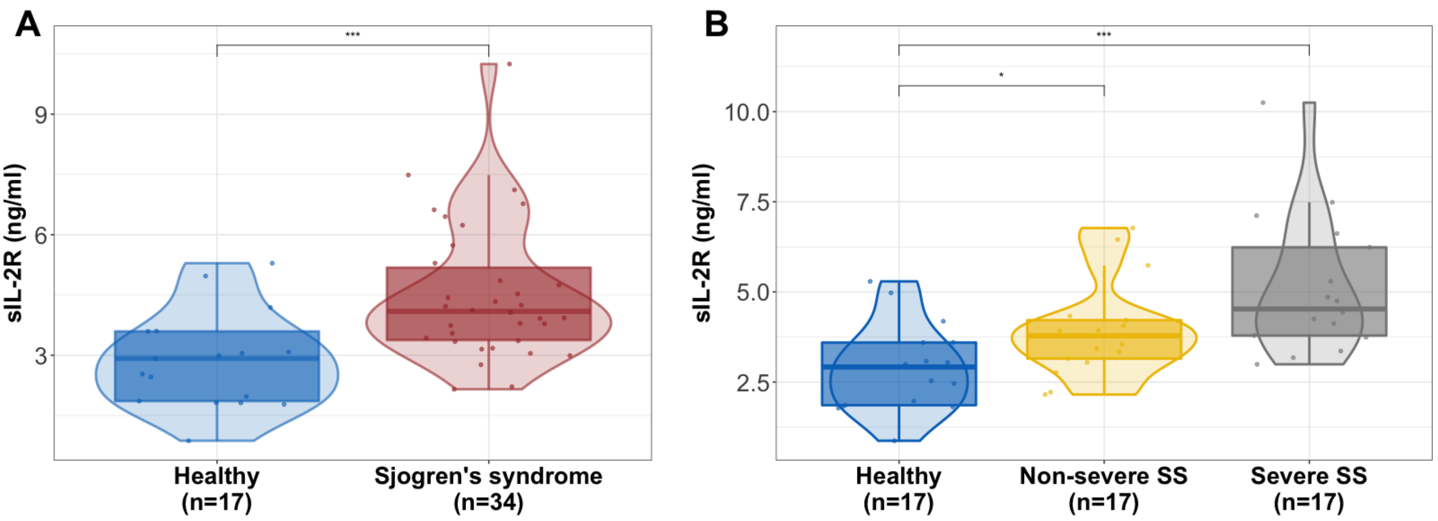

Figure S3 The selected 51 individuals for the phospho-flow cytometry analysis are representative of the whole cohort and display significantly higher sIL-2R in patients with Sjögren's syndrome (SS) **(A)**, particularly those with low saliva production **(B)**. (Mann-Whitney U test was used in the comparison between the different groups. *p ≤0.05, **p≤0.01, ***p≤0.001, and ***p≤0.0001).

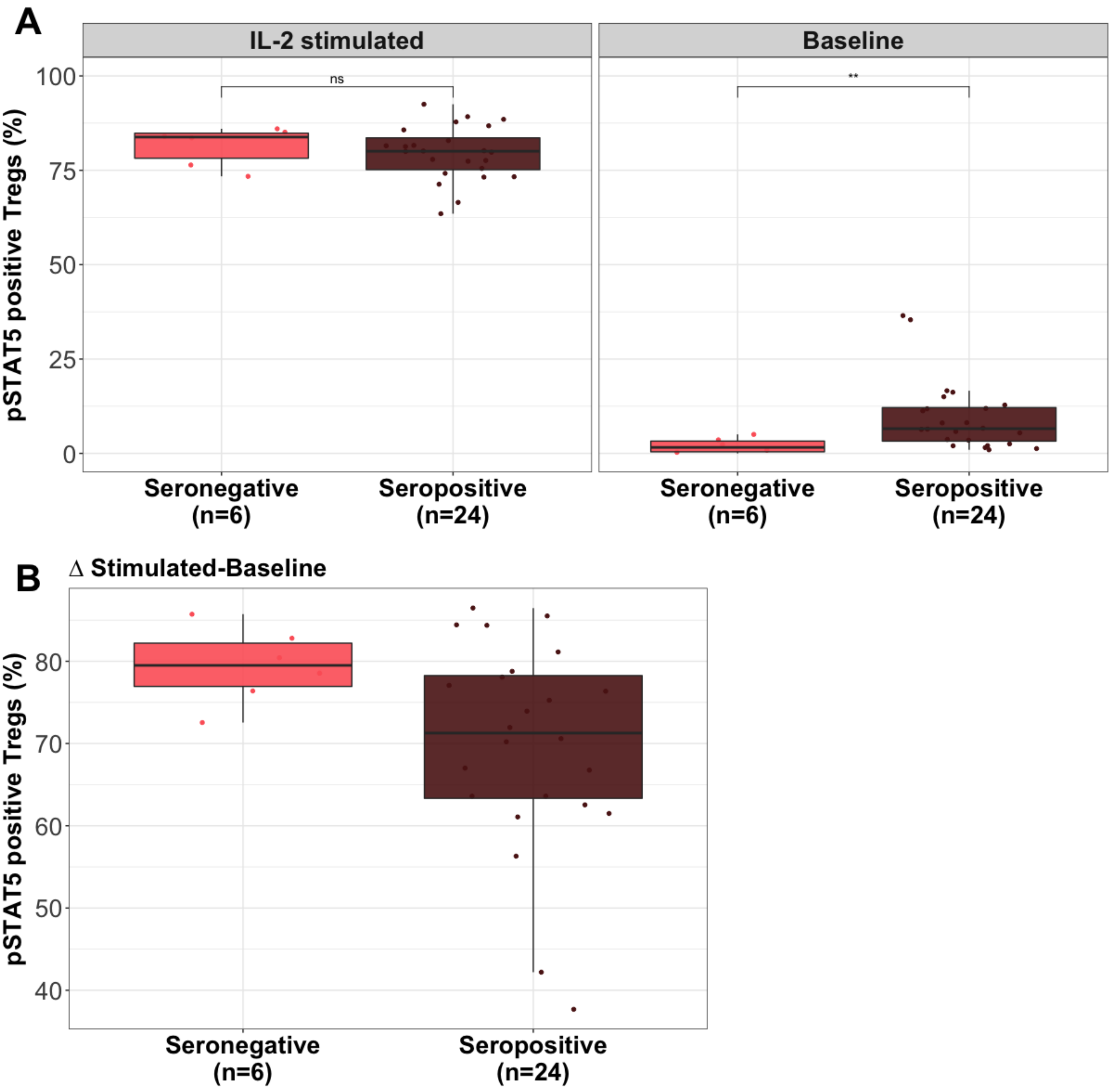

Figure S4 Percentage of pSTAT5⁺ Tregs and their association with serology. **(A)** Seropositive patients with Sjögren's syndrome had a significantly higher frequency of pSTAT5⁺ Tregs at baseline compared with seronegative patients (right panel), whereas no difference was observed in IL-2 stimulated Tregs (left panel). **(B)** The difference in pSTAT5⁺ Tregs between baseline and IL-2 stimulation was slightly lower in seropositive patients with Sjögren's syndrome. (Mann-Whitney U test was used in the comparison between the different groups. *p≤ 0.05, **p≤0.01, ***p≤0.001, and ***p≤0.0001.).

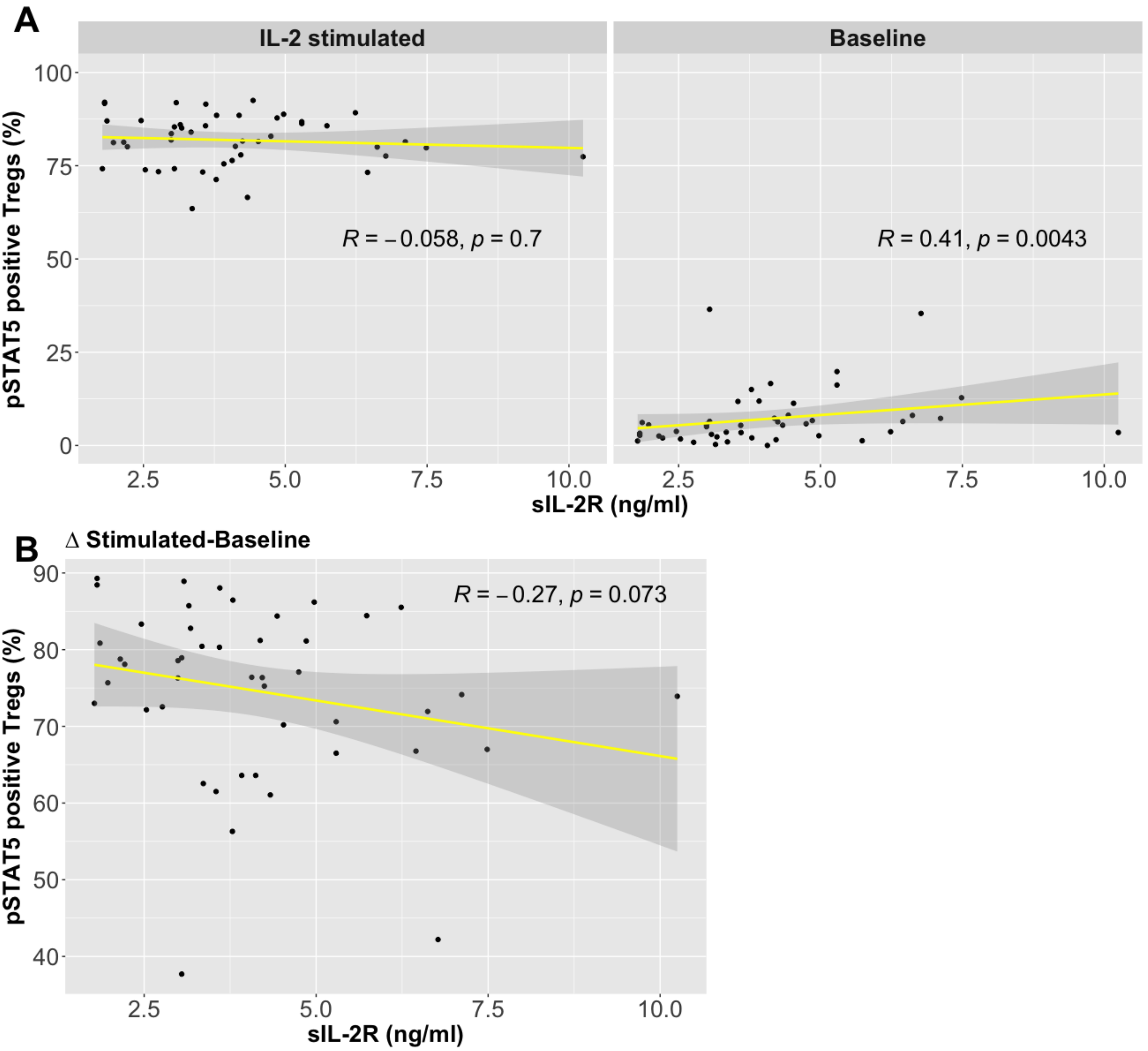

Figure S5 Frequency of pSTAT5⁺ Tregs and their association with plasma sIL-2R. **(A)** Higher plasma sIL-2R levels significantly associated with higher frequency of pSTAT5⁺ Tregs at baseline (right panel), whereas no correlation was observed in IL-2 stimulated Tregs (left panel). **(B)** The difference in pSTAT5⁺ Tregs between baseline and IL-2 stimulation did not correlate with sIL-2R (To evaluate the association between two variables we applied the Pearson correlation formula.).
